# Acute recurrent rhabdomyolysis in a Chinese boy associated with a novel compound heterozygous *LPIN1* variant: a case report

**DOI:** 10.1186/s12883-021-02050-w

**Published:** 2021-01-29

**Authors:** Ke Tong, Geng-Sheng Yu

**Affiliations:** 1 Department of Cardiovascular Disease, Children’s Hospital of Chongqing Medical University, 136 Zhongshan 2nd Road, Yuzhong District, Chongqing, 400014 China; 2 Ministry of Education Key Laboratory of Child Development and Disorders, 136 Zhongshan 2nd Road, Yuzhong District, Chongqing, 400014 China; 3 National Clinical Research Center for Child Health and Disorders (Chongqing), 136 Zhongshan 2nd Road, Yuzhong District, Chongqing, 400014 China; 4 China International Science and Technology Cooperation Base of Child Development and Critical Disorders, 136 Zhongshan 2nd Road, Yuzhong District, Chongqing, 400014 China; 5 Chongqing Key Laboratory of Pediatrics, 136 Zhongshan 2nd Road, Yuzhong District, Chongqing, 400014 China

**Keywords:** Acute recurrent rhabdomyolysis, LPIN1 deficiency, *LPIN1* gene, Novel variants, Child

## Abstract

**Background:**

*LPIN1*-related acute recurrent rhabdomyolysis (RM), first reported in 2008, is an autosomal recessive inherited metabolic disease. In recent years, *LPIN1* gene variants have been identified as one of the main causes of severe RM in children in Western countries. The disease is extremely rare in China, and we report a case of acute recurrent RM caused by a novel compound heterozygous *LPIN1* variant.

**Case presentation:**

A 15-year-old Chinese boy presented with myalgia after strenuous exercise, accompanied by transient increases in serum creatine kinase and myoglobin and persistent hyperuricaemia and hyperbilirubinaemia. Genetic analysis using high-throughput genomic sequencing and Sanger sequencing revealed that there was a compound heterozygous variant in the *LPIN1* gene of the proband: the paternal c.2047A > G(p.I683V) was an unreported missense variant, and the maternal c.2107_2108 insAGG(p.Q703delin sQE) was an unreported in-frame variant.

**Conclusions:**

In children with RM, *LPIN1* variants should always be considered in the differential diagnosis. The clinical features of our case are atypical, which highlights the importance of an accurate diagnosis by genetic testing. If detected early, the condition may be controlled, and the prognosis may be improved.

## Background

Rhabdomyolysis (RM) is a clinical entity characterized by the destruction of skeletal muscle with the resultant release of intracellular content into the bloodstream that leads to systemic complications [1]. The classic presentation of this condition is myalgia, transient muscle weakness, pigmenturia, and a marked elevation of serum creatine kinase (CK) five to ten times above the upper limit of normal serum levels [2]. The incidence of RM is low, and recurrence is even rarer. As far as we know, the recurrent RM in children is predominantly due to genetic metabolic muscle diseases [3]. Michot et al. [4] determined that variants in the *LPIN1* gene encoding LPIN1 are the second most common cause of early childhood recurrence and severe RM, second only to mitochondrial fatty acid ß-oxidation defects (FAOs). LPIN1 is an intracellular protein that controls metabolism by acting at multiple regulatory levels. This soluble protein acts at the endoplasmic reticulum to dephosphorylate phosphatidic acid (PA) to form diacylglycerol (DAG), penultimate steps in Kennedy pathway of triacylglycerol (TAG) synthesis. LPIN1 also acts in the nucleus to directly interact with DNA-bound transcription factors to regulate gene expression [5]. *LPIN1*-related acute recurrent RM was first reported by Zeharia et al. in 2008. It is an autosomal recessive inherited metabolic disease that is most commonly triggered by fever, exercise, fasting, and anaesthesia. Severe RM can cause acute kidney failure and may lead to death [6]. To the best of our knowledge, we report a case of a Chinese boy with acute recurrent RM caused by a novel compound heterozygous variant in *LPIN1* whose gene variant points are different from previous cases.

## Case presentation

A 14-year-old boy went to the hospital for a health examination, which detected a slight increase in bilirubin that was followed up regularly. Then, the boy suffered from an intermittent fever, mainly a moderate-to-high fever, lasting for 1 week. After a health examination, we found that the boy’s levels of CK, myoglobin, bilirubin and uric acid were all significantly increased. Serum biochemical test results are shown in Table 1. Physical examination showed that his body mass index was 22.73 kg/m^2^ (reference: 17.1–23 kg/m^2^), and muscular tone and power in both lower extremities were normal. We suspected this was as characteristic of RM. Then, the boy was advised to take oral sodium bicarbonate to alkalize urine, fructose and vitamin C to improve metabolism and promote cell repair until another health examination 1 month later. We found that the boy’s levels of CK, myoglobin, bilirubin and uric acid CK were higher than the previous levels. The results of these serum biochemical test are also shown in Table 1. This was the highest CK level and myoglobin level we had detected so far. His vital signs were stable, and although he had no discomfort at rest, discomfort manifested as calf myalgia after strenuous exercise. Immune function, abdominal and cardiac ultrasound, dynamic electrocardiogram, electromyography, and calf muscle MRI were all unremarkable. At the age of 12, he joined the school’s athletics team, and at the age of 13, he joined the school’s tennis team. Appropriate exercise can be tolerated by the boy. When experiencing high-intensity exercise, the boy had myalgia throughout the body. To cope with the physical education test, the boy performed high-intensity long jump training every day. However, after 5–6 long jumps, the boy began to experience fatigue, low back pain, calf pain and dark urine, while this kind of signs did not manifest among his companions. After he sat down and rested for a while, his symptoms were relieved. The parents claimed that when their child was 13 years old, he suffered from leg pain during the night-time. At that time, there were protein and red blood cells in the urinalysis, and the urine was light red. Unfortunately, no further examination was performed at that time. The boy had hyperbilirubinaemia and hyperuricaemia since the age of 14 because of high-intensity long jump training, while CK and myoglobin levels were normal in between episodes. Sometimes, there was a small amount of red blood cells and protein appearing in the urinalysis, and a slight increase in serum creatinine. Strangely, the boy underwent a bronchoscopy under general anesthesia at the age of 12 due to mycoplasma pneumonia, prior to diagnosis and this did not trigger an episode of RM. He had no chronic diseases and was not on any long-term oral medications. There was no family history of RM or any other musculoskeletal disease. His parents were both Chinese and non-consanguineous. His father was obese and had fatty liver, hyperbilirubinaemia and hyperuricaemia, but he had never experienced myalgia. His mother suffered from acute nephritis when she was young. Considering the risk of anaesthesia and invasive nature of the procedure, the parents of the boy did not give consent for the muscle biopsy.

**Table 1:** Biochemical findings of the patient

Serum Biochemistry	2019.08.28	reference	2019.09.22	reference
ALT (U/L)	26.3	0–50	13	0–40
AST (U/L)	32.3	0–55	31	0–45
CK (U/L)	1079	40–300	2610	40–300
CK-MB (ug/L)	1.11	0.21–5	1.86	0.21–5
LDH (U/L)	200	110–330	263	110–330
Myoglobin (ug/L)	203.23	<110	551.42	<110
cTnI (ug/L)	0	<0.06	0	<0.06
Cr (umol/L)	61	15.4–90.4	59	14–60
Urea (mmol/L)	4.64	2.42–6.72	3.6	2.2–7.14
TBIL (umol/L)	21.9	0–20.5	23	0–21
DBIL (umol/L)	0	0–6.7	7.5	0–6.7
IBIL (umol/L)	21.9	0–19.5	15.5	0–14
Urca (umol/L)	532.1	140–390	466	100–410
ALP (U/L)	95.4	95–405	125.1	100–360

Table legend: The table represented the results of two serum biochemical tests performed by the boy during RM. Abbreviations: *ALT* alanine aminotransferase, *AST* aspartate transaminas, *CK* creatine kinase, *CK-MB* creatine kinase myocardia band, *LDH* lactate dehydrogenase, *cTnI* cardiac troponin I, *Cr* creatinine, *TBIL* total bilirubin, *DBIL* direct bilirubin, *IBIL* indirect bilirubin, *Urca* uric acid, *ALP a*lkaline phosphatase

**Fig. 1:**
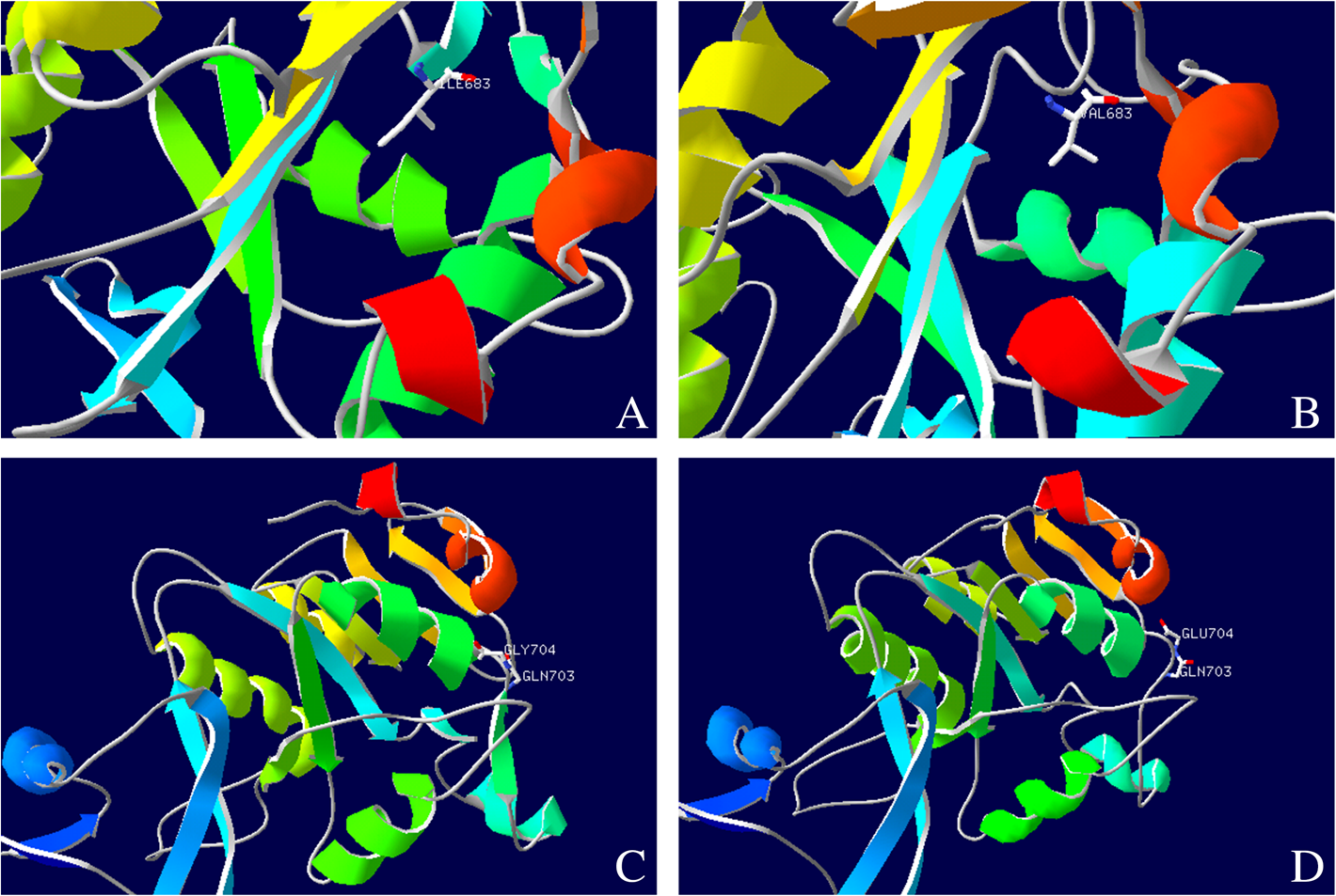
Predicted three-dimensional structure of protein at two variant points of *LPIN1* gene. **a**: Wild-type protein of the 683rd position is isoleucine; **b**: c.2047A > G variant protein becomes valine; **c**: Three-dimensional structure of wild-type protein; **d**: c.2107_2108 insAGG variant protein structure. *WT* wild type

**Fig. 2:**
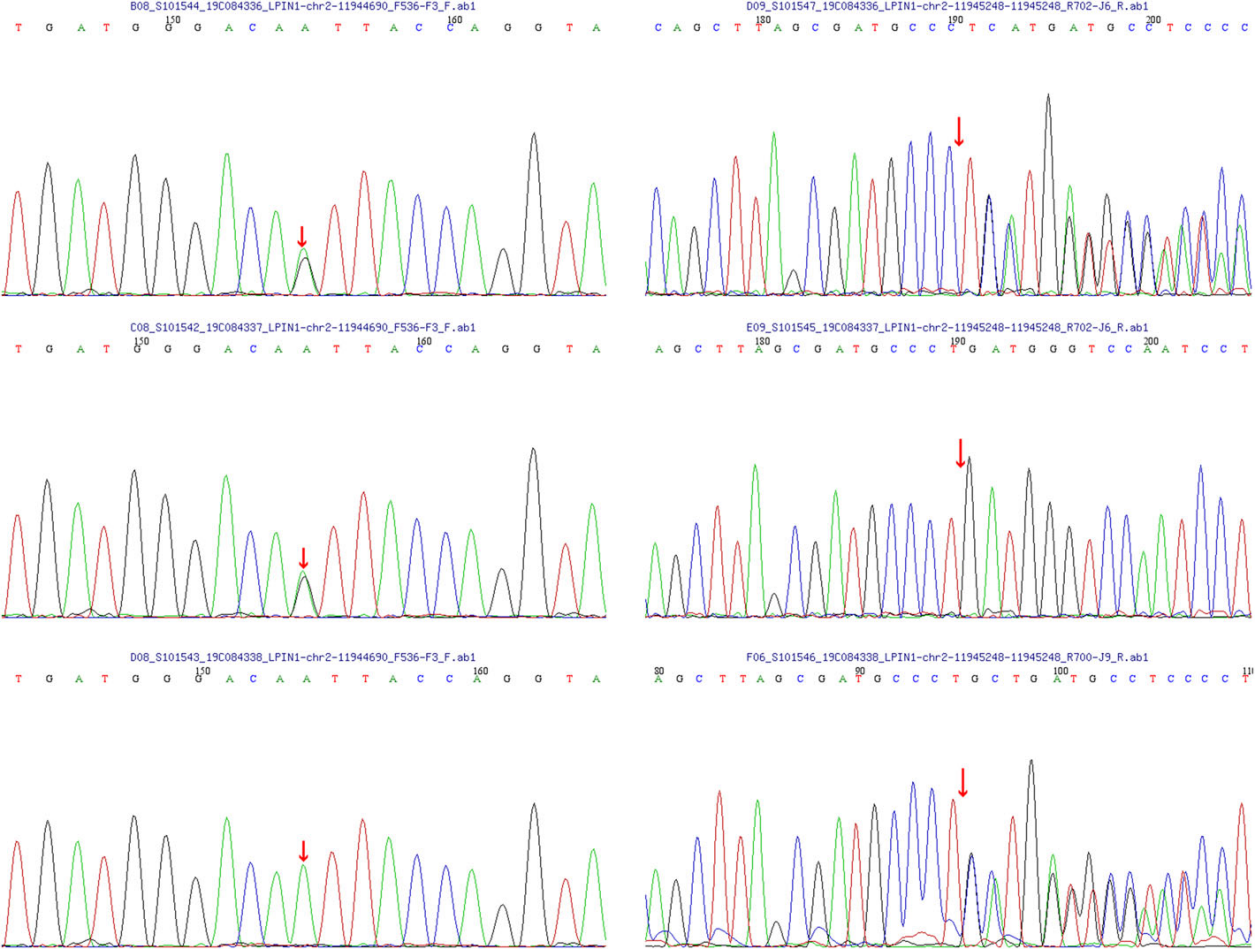
Direct sequencing showing two alleles of the proband and his parents, respectively. The proband is a compound heterozygote of c.2047A > G and p.Q703delin sQE. The first variant is from the father, and the latter is from the mother. The arrow shows the variant point. *P* proband, *F* father, *M* mother, *WT* wild type

Peripheral blood samples from the proband and the parents were collected and high-throughput genomic sequencing (next-generation sequencing) of 233 genes related to neuromuscular diseases (Table 2) was performed by Beijing Mygenostics Co. Ltd. Informed consent was obtained from the proband and his guardian for gene detection. The results showed that the *LPIN1* gene variants in this proband included the missense variant c.2047A > G(p.I683V) and in-frame variant c.2107_2108 insAGG(p.Q703delin sQE). The c.2047A > G variant of the 2047th nucleotide in the coding region from adenine to guanine results in the 683rd amino acid changing from isoleucine to valine; c.2107_2108 insAGG changed by way of indels, and the 703rd amino acid changed from glutamine (Q) to glutamine (Q) and glutamate (E), which caused the protein length to change (Fig. 1). Sanger sequencing confirmed that the proband had compound heterozygous *LPIN1* variants; the former was from his father, while the latter was from his mother (Fig. 2). According to the standards and guidelines of the American Society for Medical Genetics and Genomics in 2015 [7], both variants were classified as variants of uncertain significance. The frequency of the missense variant and the in-frame variant detected in our case in the normal population database was 0.00002, indicating that both were low-frequency variants (criteria: PM2), and each bioinformatics protein function prediction software predicted the missense variant to be harmful (criteria: PP3) and the other to be unknown. The in-frame variant resulted in a change in protein length caused by a small insertion in the nonrepetitive sequence region (criteria: PM4).

**Table 2:** Summary of all genes covered by the panel

*ABHD5*	*ACAD8*	*ACADL*	*ACADM*	*ACADS*	*ACADVL*	*ACTA1*	*ACVR1*
*AGK*	*AGL*	*AGRN*	*ALDOA*	*ALG13*	*ALG14*	*ALG2*	*ANO5*
*ATP2A1*	*ATP5F1A*	*B3GALNT2*	*B4GAT1*	*BAG3*	*BIN1*	*CACNA1A*	*CACNA1S*
*CAPN3*	*CAV3*	*CAVIN1*	*CCDC78*	*CFL2*	*CHAT*	*CHKB*	*CHRNA1*
*CHRNB1*	*CHRND*	*CHRNE*	*CHRNG*	*CHST14*	*CLCN1*	*CNTN1*	*COL12A1*
*COL13A1*	*COL6A1*	*COL6A2*	*COL6A3*	*COLQ*	*COQ2*	*COQ4*	*COQ6*
*COQ7*	*COQ8A*	*COQ9*	*CPT1A*	*CPT2*	*CRYAB*	*DAG1*	*DES*
*DGUOK*	*DMD*	*DNA2*	*DNAJB6*	*DNM2*	*DOK7*	*DOLK*	*DPAGT1*
*DPM1*	*DPM2*	*DYSF*	*ECEL1*	*ELP1*	*EMD*	*ENO3*	*ETFA*
*ETFB*	*ETFDH*	*ETHE1*	*FBXL4*	*FHL1*	*FKBP14*	*FKRP*	*FKTN*
*FLNA*	*FLNC*	*G6PC*	*GAA*	*GBE1*	*GFPT1*	*GMPPB*	*GNE*
*GYG1*	*GYS1*	*HACD1*	*HADH*	*HADHA*	*HADHB*	*HNRNPA1*	*HNRNPA2B1*
*HNRNPDL*	*HSPG2*	*IGHMBP2*	*ISCU*	*ISPD*	*ITGA7*	*ITGA9*	*KBTBD13*
*KCNA1*	*KCNE3*	*KCNE4*	*KCNH2*	*KCNJ16*	*KCNJ2*	*KCNQ1*	*KIF21A*
*KLHL40*	*KLHL41*	*KLHL9*	*LAMA2*	*LAMB2*	*LAMP2*	*LARGE1*	*LDB3*
*LDHA*	*LMNA*	*LMOD3*	*LPIN1*	*LRP4*	*MAMLD1*	*MATR3*	*MEGF10*
*MGME1*	*MLYCD*	*MPV17*	*MSTN*	*MTM1*	*MTMR14*	*MTTP*	*MUSK*
*MYBPC1*	*MYBPC3*	*MYF6*	*MYH14*	*MYH2*	*MYH3*	*MYH7*	*MYH8*
*MYLK2*	*MYO9A*	*MYOT*	*NDUFB3*	*NEB*	*OPA1*	*PABPN1*	*PDSS1*
*PDSS2*	*PFKM*	*PGAM2*	*PGK1*	*PGM1*	*PHKA1*	*PHOX2A*	*PIEZO2*
*PLEC*	*PLOD1*	*PLOD2*	*PLOD3*	*PMM2*	*PNPLA2*	*POLG*	*POLG2*
*POMGNT1*	*POMGNT2*	*POMK*	*POMT1*	*POMT2*	*PREPL*	*PRKAG2*	*PUS1*
*PYGM*	*RAPSN*	*RBCK1*	*RRM2B*	*RXYLT1*	*RYR1*	*SCN4A*	*SCN5A*
*SELENON*	*SGCA*	*SGCB*	*SGCD*	*SGCE*	*SGCG*	*SIL1*	*SLC16A2*
*SLC22A5*	*SLC25A1*	*SLC25A20*	*SLC25A4*	*SLC37A4*	*SLC3A1*	*SLC5A7*	*SNAP25*
*SPAST*	*SPEG*	*STIM1*	*SUCLA2*	*SUCLG1*	*SYNE1*	*SYNE2*	*SYT2*
*TCAP*	*TIA1*	*TK2*	*TMEM43*	*TNNI2*	*TNNT1*	*TNNT3*	*TNPO3*
*TOR1A*	*TOR1AIP1*	*TPM2*	*TPM3*	*TRAPPC11*	*TRIM32*	*TTN*	*TTR*
*TUBB3*	*TWNK*	*TYMP*	*VCP*	*VPS13A*	*XK*	*YARS2*	*ZBTB42*
*ZC4H2*

Although the result of genetic testing was uncertain regarding pathogenicity, we communicated the possibility of *LPIN1*-related RM to the parents and recommended that the boy restrict strenuous exercise, have proper rest, drink adequate water, supplement enough energy and take oral sodium bicarbonate (0.5 g/time, 3 times/day) for urine alkalization when myalgia and dark urine occur. Under our guidance, his daily life and studies have not been affected.

## Discussion and conclusions

*LPIN1*-related acute recurrent RM is a life-threatening disease that is characterized by the age of the first onset being younger than 6 years old and the peak CK level at the onset exceeding 10,000 IU/L [8]. We discovered a novel compound heterozygous variant in the *LPIN1* gene (p.I683V and p.Q703delin sQE) in a Chinese boy with acute recurrent RM, and his clinical characteristics are quite different from those reported in previous studies. Our proband is a 15-year-old adolescent with a later age of onset and only one episode of mild RM. His serum CK and myoglobin were normal in between episodes, but there was long-term hyperuricaemia and hyperbilirubinaemia. To the best of our knowledge, we report the third case of *LPIN1*-related acute recurrent RM in a patient of Chinese ethnicity, while the case with hyperuricaemia and hyperbilirubinaemia is shared for the first time (MIM#268200).

We searched the PubMed database using “acute recurrent rhabdomyolysis” and “*LPIN1*” as keywords. There were 57 patients (including our case) with complete clinical data who were genetically diagnosed with acute recurrent RM caused by the *LPIN1* gene variant [4, 6, 9–22]. These patients were reported from 18 different countries, of which only 3 cases were of Chinese ethnicity. The findings are presented in Table 3. We found that *LPIN1*-related acute recurrent RM was more common in males than in females (28/57 49.1% vs. 24/57 42.1%). The first onset was before the age of 6 for the most part, and the number of episodes was usually 3 or fewer. The peak CK level was often greater than 10*10^4^U/L. In 40 cases, potential trigger factors had been reported, among which fever was the most common (26/40 65.0%) ([4, 6, 11, 13, 15, 16, 20, 22], our case), followed by infection (12/40 30.0%) [11, 13–16, 19–21], fasting (7/40 17.5%) [4, 16], anaesthesia (5/40 12.5%) [4, 14], and exercise or physical activity (4/40 10.0%) [11, 14, 16, 17]. Only 9 cases (9/57 15.8%) [6, 9, 11, 13, 17–19, 22] underwent muscle biopsy, which is an invasive procedure that seems to be unpopular. There were 8 deaths (8/57 14.0%) [4, 9, 13], suggesting a high mortality rate of this disease. We also summarized the variant points of 56 cases whose genetic results were reported and finally found 38 different variant points [4, 6, 9–23], including missense variants, frameshift variants, in-frame variants, nonsense variants, and exon deletions. The variant points are summarized in Table 4. There were 32 cases with homozygous variants (32/56 57.1%) and 24 cases with compound heterozygous variants (24/56 42.9%). There were 6 variant points in Chinese patients, all of which were compound heterozygous variants; c.2047A > G(p.I683V), c.2107_2108 insAGG(p.Q703delin sQE), c.2428C > T(p.Arg810Cys) and c.1949_1967dupGTGTCACCACGCAGTACCA (p.Gly657CysfsX12) were only detected in individuals of Chinese ethnicity ([15, 16], our case), and these might be Chinese ethnicity-specific variants.

**Table 3:** Summary of clinical information of 57 patients with *LPIN1*-related acute recurrent rhabdomyolysis

Ethnic origin	Numberof cases	Sex			Age at onset				Number ofepisodes		CK peak UI/L				Number ofmuscle biopsies	Numberof deaths
		M	F	NM	<6Y	6 ~ 16Y	>16Y	NM	≤3	>3	<1*10^4^	1*10^4~^10*10^4^	>10*10^4^	NM		
France [4, 6, 9, 10]	19	9	8	2	10	1	0	8	10	9	0	5	14	0	2	3
Jordan [13]	8	5	3	0	7	1	0	0	7	1	0	5	3	0	1	1
Austria [14]	5	2	3	0	4	1	0	0	4	1	0	0	5	0	0	0
China ([15, 16],our case)	3	3	0	0	2	1	0	0	3	0	1	0	2	0	0	0
United Kingdom[4, 17, 18]	3	1	2	0	1	1	1	0	2	1	0	0	3	0	2	1
Belgium [4]	3	2	1	0	3	0	0	0	3	0	0	2	1	0	0	2
North Africa [4]	2	1	1	0	2	0	0	0	2	0	0	1	1	0	0	0
Germany [4]	2	0	2	0	2	0	0	0	1	1	0	0	2	0	0	0
Mauritania [4, 6]	2	1	0	1	2	0	0	0	2	0	0	0	2	0	0	0
America [11, 12]	2	1	1	0	2	0	0	0	2	0	0	0	2	0	1	0
Arab [6]	1	1	0	0	1	0	0	0	1	0	0	0	1	0	1	0
Egypt [4]	1	1	0	0	1	0	0	0	1	0	0	1	0	0	0	1
Ireland [19]	1	0	1	0	1	0	0	0	0	0	0	0	1	0	1	0
Palestine [7]	1	0	0	1	1	0	0	0	1	0	0	1	0	0	0	0
Canada [20]	1	1	0	0	1	0	0	0	0	1	0	0	1	0	0	0
Portugal [21]	1	0	0	1	1	0	0	0	1	0	0	0	0	1	0	0
Sri Lanka [22]	1	0	1	0	1	0	0	0	0	1	0	1	0	0	1	0
Italy [18]	1	0	1	0	0	0	1	0	1	0	0	0	1	0	0	0
Total (%)	57	28/57(49.1)	24/57(42.1)	5/57(8.8)	42/57(73.7)	5/57(8.8)	2/57(3.5)	8/57(14.0)	41/57(71.9)	15/57(26.3)	1/57(1.8)	16/57(28.1)	39/57(68.4)	1/57(1.8)	9/57(15.8)	8/57(14.0)

*M* male, *F* female, *CK* creatine kinase, *Y* year, *NM* not mentioned

**Table 4:** Summary of gene variant points

Variant	Amino Acid Change	Ethnic origin	Variant	Amino Acid Change	Ethnic origin
c.2047A > G	p.I683V	China[our case]	c.944C > G	p.Ser315X	France [9], France-Asia [4]
c.2107_2108 insAGG	p.Q703delinsQE	China[our case]	c.643G > T	p.E215X	Arab [6]
c.2295-866_2410-30del	p.Glu766_Ser838del	France [9], Belgium [4], United Kingdom [4, 18], Germany [4], Ireland [19], Austria [14], Italy [18]	c.2398C > T	p.R800X	France [6]
c.1162C > T	p.Arg388X	France [9], France-Asia [4], North Africa [4], Palestine [6], China [16], Jordan [13]	c.297 + 2 T > C	NM	Mauritania、France [6]
NM	p.Asn417LysfsX22	France [10]	c.1949_1967dupGTGTCACCACGCAGTACCA	p.Gly657CysfsX12	China [15]
NM	p.Cys30LeufsX3	France [10]	c.2410G > C	p.Asp804His	China [15]、United Kingdom [17]
c.192 + 2 T > C	p.Cys30LeufsX3	Mauritania [4]	C.942C > A	p.Cys314X	Ireland [19]
c.2401C > T	p.Arg801X	France [4]	c.1904 T > C	p.Leu635Pro	America [11]
c.1441 + 2 T > C	p.Asn417LysfsX22	France [4]	NM	E766-S838_del	America [11]
c.377_380dup	p.Met128GlnfsX45	France [4]	c.2428C > T	p.Arg810Cys	China [16]
c.2513 + 1G > A	p.Asp804ValfsX6	Egypt [4]	c.1255_1256dupGA	p.Asp419fs	Austria [14]
c.921delT	p.Gln308ArgfsX36	United Kingdom [4]	c.394_409del	p.Asp132fs	Austria [14]
c.946_952del	p.Asp316LeufsX26	Germany [4]	c.2174G > A	p.Arg725His	Jordan [13]
c.1259delC	p.Pro420LeufsX39	France [4]	c.2395G > C	p.Gly799Arg	Jordan [13]
c.2513 + 1G > A	p.Asp804ValfsX6	France [4]	c.1684G > T	p.Glu562X	Sri Lanka [22]
c.2253_2254del	p.Leu752AlafsX17	Germany [4]	NM	p.Arg61X	NM [23]
c.57C > A	p.Tyr19X	Germany [4]	NM	p.Asp170X	NM [23]
c.2142-2A > G	NM	Portugal [21]	NM	p.Ala227GlyfsX2	NM [23]
c.1381delC	p.Leu461SerfsX47	Canada [20]	NM	p.Glu477X	NM [23]

*NM* not mentioned

The LPIN protein family is composed of three members, LPIN1, LPIN2, and LPIN3, encoded by the *LPIN1* gene, *LPIN2* gene, and *LPIN3* gene, each of which is 100 kDa in size and has 44–48% amino acid similarity. They are Mg^2+^-dependent phosphatidic acid phosphatase (PAP) enzymes that catalyse a key reaction in glycerolipid biosynthesis [24]. Only LPIN1 is expressed in skeletal muscle and plays a key role in human muscle function [25]. The *LPIN1* gene is located on chromosome 2p25.1 with two highly conserved domains: the N-terminal LPIN (N-LIP) region spans the first 108 residues, and the C-terminal LPIN (C-LIP) domain consists of 624–830 amino acids. The C-LIP domain contains two key domains: the PAP catalytic motif Asp-Xaa-Asp-Xaa-Thr (DXDXT (689–693)) and the transcriptional coactivator motif Leu-Xaa-Xaa-Ile-Leu (LXXIL (678–682)) [16, 26]. They both play an important role in the activity of PAP. We discovered a novel missense variant c.2047A > G(p.I683V) and a novel in-frame variant c.2107_2108 insAGG(p.Q703delin sQE). The onset of our case did not occur until adolescence, and the cases of delayed disease onset have been reported before [17–19, 22]. The peak CK level was only 2610 U/L, which was significantly different from previously reported cases. The reason for the delayed disease onset and mild phenotype may be due to the existence of novel missense variants and in-frame variants rather than loss-of-function truncating variants. Schweitzer et al. [11] found that single amino acid substitutions in human LPIN1 disrupt PAP activity but may not always affect nuclear transcriptional regulator function, and truncating variants (such as frameshift, nonsense and deletion) will cause loss of protein function. Therefore, the novel variants in the C-LIP domain we discovered may have a much smaller impact than the truncating variants with direct loss of function. In addition, LPIN2 and LPIN3 share a similar structure with LPIN1, and we speculate their presence can compensate for the lack of LPIN1. However, the relationship between expression patterns and the compensatory roles of LPIN2 and LPIN3 in skeletal muscle with LPIN1 deficiency is unclear, and further study is necessary. Similarly, environmental exposure may also have a regulatory effect on the occurrence of RM [18]. However, a clear limitation of the present report is that definitive pathogenicity of the missense variant and the in-frame variant could not be established due to lack of experimental evidence at the protein level. Future studies are therefore warranted to clarify the significance of c.2047A > G and c.2107_2108 insAGG in *LPIN1*-related RM.

LPIN1 has transcriptional coregulatory activity, which, through association with peroxisome proliferator-activated receptor alpha (PPARα) and peroxisome proliferator-activated receptor gamma coactivator 1-alpha (PGC-1α), regulates lipid metabolism and the mitochondrial respiratory chain (mRC) [27]. Despite the well-known physiological roles of LPIN1 in lipid biosynthesis and transcriptional regulation in adipocytes and the liver, the pathophysiological mechanisms leading to muscle dissolution remain to be elucidated. Fever and intense effort lead to high temperatures and high circulating levels of proinflammatory mediators such as cytokines and chemokines [24]. In addition, the proinflammatory stress induced by the combination of tumor necrosis factor alpha and Interleukin-1 beta (TNFα+IL-1β) intensifies the accumulation of lipid droplets (LDs), decreases the activity of carnitine palmitoyltransferase 1 (CPT1) and increases the content of triacylglycerol, highlighting the crucial role of inflammation in the pathogenesis of LPIN1 deficiency [27]. Zhang et al. [5] found that in the case of LPIN1 deficiency, a blockade in PAP activity leads to reduced DAG levels and impaired activation of the protein kinase D (PKD)-Vps34 signalling cascade in autophagy clearance, preventing normal maturation of autolysosomes. These defects may lead to the RM observed in LPIN1 deficiency. Interestingly, our case showed normal muscle function between episodes of RM, which indicated that mitochondrial function was sufficient to meet the energy requirements of daily activities. Rashid et al. [28] revealed that LPIN1 deficiency caused severe sarcoplasmic reticulum (SR) stress, leading to the activation of lipogenic sterol regulatory element binding protein 1c (SREBP1c)/SREBP2 factors, the accumulation of fibroblast growth factor 21 (Fgf21) cytokines, and alterations in SR mitochondrial morphology, which were contributing factors to the myopathy associated with LPIN1 deficiency.

Pelosi et al. [29] found that the adipose tissue of patients with *LPIN1* gene variants showed a significant decrease in LPIN1 levels and PAP activity, but the adipose tissue appeared to develop normally and had normal lipid reserves. The levels of metabolic components such as triglycerides and cholesterol remained unremarkable in the serum in our case, consistent with the results of previous studies. Our patient underwent muscle-related examinations, such as electromyography and calf muscle MRI, and the results were not significant, consistent with the report of Minton et al. [17], which showed that the skeletal muscle morphology of patients with LPIN1 deficiency can be insignificant. It is worth noting that our patient had persistent hyperuricaemia since the first episode of RM. We believe that a large number of damaged muscle cells may release endogenous purines, which are metabolized into uric acid, thereby increasing uric acid production and leading to hyperuricaemia. Too much uric acid may be further concentrated and deposited in the renal tubules, which may aggravate the obstruction caused by myoglobin and even cause renal dysfunction [30]. We speculate that hyperuricaemia may increase the risk of renal failure in RM. If detected early, the condition may be controlled, and the prognosis may be improved. In addition, our patient also had a continuous mild increase in bilirubin. Serum bilirubin has antioxidant and anti-inflammatory effects, which can inhibit kidney damage to a certain extent [31]. High serum total bilirubin levels are independently associated with a high glomerular filtration rate, and those with high serum total bilirubin levels have a lower risk of renal damage [32]. Because of normal kidney function in our patient, we speculate that the mild increase in bilirubin is protective to delay the appearance of kidney damage, although it is difficult to know in our patient whether it is associated with the onset of *LPIN1*-associated RM. Moreover, it should be noted that our case is easily confused with phosphoglycerate kinase (PGK) deficiency, which is a rare cause of congenital hemolytic anemia accompanied by hyperbilirubinaemia, muscle weakness, myalgia and myoglobinuria [33]. Although our case has similar clinical features with PGK deficiency, it has been ruled out by genetic testing.

Our patient has been taking supportive treatments at home, such as rest, rehydration, energy supplementation, a low-purine diet, and supplements to alkalize urine, and he is living well at present. Symptomatic treatment of RM with LPIN1 deficiency mainly includes early detection, active hydration, high-energy carbohydrate intake, and monitoring of hyperkalaemia and arrhythmia [19]. To avoid long-term complications, it is essential to prevent fever, restrict strenuous exercise, and seek medical attention in cases of myalgia. Moreover, Pichler et al. [14] found that five patients with *LPIN1*-related RM were advised to use high-concentration glucose solutions early to provide calories, which reduced the duration of RM from the typical 7–10 days to 5 days. Meijer et al. [20] successfully used dexamethasone, which can stimulate LPIN1 expression and PAP activity as an inducer of PGC-1α, to treat a patient diagnosed with *LPIN1* variants. However, whether high-concentration glucose solutions and dexamethasone can effectively treat RM remains to be further explored in the future.

In summary, we diagnosed a Chinese boy with acute recurrent RM and discovered the novel compound heterozygous variants c.2047A > G(p.I683V) and c.2107_2108 insAGG (p.Q703delin sQE). According to a systematic literature review, *LPIN1*-related acute recurrent RM is more common in males. The first onset is before the age of 6, and the number of episodes is usually within 3. The peak CK level is often greater than 10*10^4^U/L. Fever, infection and fasting are the most common trigger events, and gene variants are common as homozygous variants, while the Chinese cases being reported all involve compound heterozygous variants. As the mortality rate due to LPIN1 deficiency is as high as 14.0% and some patients have atypical symptoms, as in our case, it highlights that paediatric clinicians in the Departments of Cardiology, Neurology, Nephrology, and Immunology should promptly detect the disease and treat it symptomatically. Genetic testing, a method to clearly diagnose and analyse the source of pathogenic genes, is increasingly recognized. Our research is of reference value for an in-depth understanding of the clinical and laboratory characteristics of *LPIN1*-related acute recurrent RM and for improving the early diagnosis and clinical management of patients with this disease.

## Data Availability

The data used to support the findings of this study are available from the corresponding author upon request, without breaching participant confidentiality.

## Abbreviations

RM: Rhabdomyolysis
CK: Creatine kinase
DAG: Diacylglycerol
TAG: Triacylglycerol
PA: Phosphatidic acid
HGMD: The Human Gene Mutation Database
PAP: Phosphatidic acid phosphatase
N-LIP: The N-terminal LPIN
C-LIP: The C-terminal LPIN
PKD: The protein kinase D
SR: Sarcoplasmic reticulum
PGC-1α: Peroxisome proliferator activated receptor gamma coactivator 1-alpha
PGK: Phosphoglycerate kinase
TNFα: Tumor necrosis factor alpha
IL-1β: Interleukin-1 beta
